# Quantification of play behaviour in calves using automated ultra-wideband location data and its association with age, weaning and health status

**DOI:** 10.1038/s41598-024-59142-z

**Published:** 2024-04-17

**Authors:** J. A. Vázquez-Diosdado, C. Doidge, E. V. Bushby, F. Occhiuto, J. Kaler

**Affiliations:** School of Veterinary Medicine and Science, Sutton Bonington Campus, University of Nottingham, Leicestershire, LE12 5RD UK

**Keywords:** Play, Positive welfare, Calf, Machine learning, Quantification, Precision livestock technology, Animal behaviour, Behavioural ecology

## Abstract

Play behaviour can act as an indicator of positive animal welfare. Previous attempts to predict play behaviour in farmed calves are limited because of the classification methods used, which lead to overestimation, and the short time periods that calves are observed. The study aimed to automatically classify and quantify play behaviour in farmed calves using location data from ultra-wide band sensors and to investigate factors associated with play behaviour. Location data were collected from 46 calves in three cohorts for a period of 18 weeks. Behavioural observations from video footage were merged with location data to obtain a total of 101.36 h of labelled data. An AdaBoost ensemble learning algorithm was implemented to classify play behaviour. To account for overestimation, generally seen in low-prevalence behaviours, an adjusted count technique was applied to the outputs of the classifier. Two generalized linear mixed models were fitted to investigate factors (e.g. age, health) associated with duration of play and number of play instances per day. Our algorithm identified play behaviour with > 94% accuracy when evaluated on the test set with no animals used for training, and 16% overestimation, which was computed based on the predicted number of samples of play versus the number of samples labelled as play on the test set. The instances and duration of play behaviour per day significantly decreased with age and sickness, whilst play behaviour significantly increased during and after weaning. The instances of play also significantly decreased as mean temperature increased. We suggest that the quantification method that we used could be used to detect and monitor other low prevalence behaviours (e.g. social grooming) from location data, including indicators of positive welfare.

## Introduction

In livestock species it is now widely understood that good welfare is not only beneficial for animal health but also for optimal production^1^. Members of the public value good animal welfare^2,3^ and animal welfare is a priority for veterinarians^4^ but they have expressed a need for research to develop better animal welfare assessment^5^. Typically, assessment of animal welfare has focused on the absence of negative experiences and emotions; however, there is increasing focus on the presence of positive emotions and experiences^6^. An animal may be in a positive welfare state when they are able to actively engage in positive interactions with their physical and social environment, for example, by playing with affiliative conspecifics. Play behaviour has been shown to occur more frequently when the animals’ needs are met and in the absence of negative stress^6,7^. In contrast, negative or stressful events such as disbudding and reduced feed allowance show reductions in play behaviour in calves^8,9^. This suggests that play behaviour can act as an indicator of positive animal welfare^10^.

Play behaviour in animals can be categorised as either social play, object play, or solitary locomotor/rotational play^11^ and varies between and within species^12,13^. In farmed calves, locomotor play behaviour has been shown to be influenced by a range of factors including age and sex^14^, nutrition and weaning^8,15^, social environment^16,17^, and health status^18^. Typically, these studies measure behaviour using video recordings scored by researchers, which can be time consuming and, as a result, limit the amount of time and the number of animals in which the behaviour can be assessed. Alternatively, precision livestock technologies provide a new opportunity to capture and measure behaviours in an automated way for a large number of individuals simultaneously over a long period of time.

Classification algorithms tend to overestimate when predicting low prevalence behaviours such as movement activity^19^, rumination^20^ and play behaviour^19^. To prevent this, we have developed a classification and adjusted count algorithm for the detection of play behaviour in calves^19^. Whilst our algorithm was useful for indicating the presence or absence of play behaviour, it was not possible to monitor play behaviour long-term and measure changes in the behaviour over time or due to internal and external factors. This was because the battery life of the accelerometer was limited due to the high sampling frequencies used. As play is a distinctive behaviour^19^, it might be possible to reduce the sampling frequency as long as the frequency remains twice the highest frequency in the signal^21^. This would extend the battery life of sensors and enable collection of extensive long-term data on play behaviour, which could provide an automated alternative to the time-consuming visual assessment of play behaviour and welfare status (Qualitative Behavioural Assessment)^22^.

Ultra-wide band (UWB) location sensors offer a means of monitoring animal behaviour by providing precise location data of individual animals with minimal human input, over longer periods of time. Although these data have been shown to perform well when predicting lying behaviour of dairy cows^23^ and were used to measure movement behaviours in calves^24^, the use of UWB location sensors in cattle is still limited. To the authors’ knowledge, location data have not been used to measure behaviours associated with positive welfare, such as locomotor play.

This study aimed to improve the prediction and monitoring of play behaviour in calves by using UWB location data in combination with machine learning classification and an adjusted count quantification algorithm. This quantification algorithm enabled us to correct for overestimation in the prediction of the classifier, which has been reported in other low-prevalence behaviours such as movement activity^25^ and rumination^19^. Then, the prediction outputs of the evaluated algorithm to identify play behaviour were used to examine how play behaviour may be impacted by factors, such as sex, breed, age, weaning and health status, in farmed calves.

## Materials and methods

### Ethics

Ethical permission for all the methods of the observational trial described was obtained for the School of Veterinary Medicine and Science, University of Nottingham (unique reference number 1481150603). All methods were performed in accordance with the relevant guidelines and regulations and are reported in accordance with the ARRIVE guidelines^26^.

### Data collection and processing

#### Animals, Housing and Farm Management.

The study took place at the Centre of Dairy Science Innovation at the University of Nottingham, UK, between the 27th of May 2021 and the 20th of December 2022. Calves were separated from the dam within 4 h of birth and housed in pairs (two calves paired together) in straw bedded pens (3 m × 2 m), as per normal farm animal management. Then, calves were moved into one of two adjacent straw-bedded pens (6 m × 10 m) to form cohorts. The cohorts were formed when there were at least 15 calves that were no less than two weeks of age available for the study. Cohort 1 was formed in June 2021, cohort 2 was formed in May 2021 and cohort 3 was formed in September 2022. A total of 46 calves from 3 cohorts of 15 to 16 calves each were included in the study, as described in Table 1.

**Table 1:** Number of calves per cohort with the sex, breed, mean age of the cohort on day 1 of the study, pen and period.

Cohort	Number of calves	Sex and breed		Average age at the start (in days)	Pen	Period
		Female HO	Male HO x AA			
1	15	12	3	34.6	A	June–August 2021
2	16	16	0	30.31	B	May–Sept 2021
3	15	15	0	35.26	A	Sept–Dec 2022

HO, Holstein–Friesian; HOxAA, Holstein–Friesian cross Aberdeen Angus.

The calves stayed in the 6 m × 10 m pens for up to 18 weeks. Each individual pen had an automatic feeder, a water trough, and a tank with concentrates. The calves were not provided with enrichment. Calves had access to concentrates, chopped straw and water ad-libitum. Ambient temperature was collected from temperature sensors located in the building (ALTA 900 MHz Industrial Humidity Sensors with Probes) and was sampled every 10 min.

In this study, calves were fed milk replacer (Milkivit Energizer ECM, Trouw Nutrition GB) from an automatic milk feeder for the entire milk-feeding period, which occurred in the first 35 days. Calves had continuous access to the feeder. Each individual calf had a Radio Frequency Identification (RFID) ear tag, which could be read by the automatic milk feeder. This allowed the automatic milk feeder to identify the calf present and dispense the corresponding allowance. During the milk-feeding period, a maximum of 2 L every 2 h up to a total daily allowance of 10 L were distributed by the automatic feeder. From day 36 of the study, the allowance was reduced by 400 ml/day until it was reduced to zero on day 60. Based on their allowance, calves were categorised as: pre-weaned (from day 1 to day 35), weaning step-down (from day 36 to day 60) and weaned (from day 60 onwards).

Calves were health monitored by a trained researcher who manually inspected all calves twice a week for signs of ill health using the Wisconsin calf health scoring system^27^. Based on the total Wisconsin score, calves were categorised as healthy if they scored from 0 to 2, moderate if their score was 3 or 4 and sick if their score was more than or equal to 5. This scoring system combined clinical examination of nasal discharge, ear score, eye score, coughing and rectal temperature score. A total of 15 calves (4 in cohort 1, 8 in cohort 2 and 3 in cohort 3) were detected as sick according to the Wisconsin score. Any calves showing signs of ill health were treated according to farm protocols and advice from the farm’s veterinary surgeon. All calves received a respiratory vaccine at 9 days of age (Rispoval RS + Pi3 IntraNasal; Zoetis).

#### Local position system

The UWB-based location system (SEWIO, Brno, Czech Republic) consisted of the UWB tags, anchor sensors, and a dedicated software (TrackLab). UWB can be defined as a radio frequency (RF) signal that has a bandwidth greater than 500 MHz^28^, which allows large amounts of data to be transmitted while consuming transmit energy^29^.

**Figure 1:**
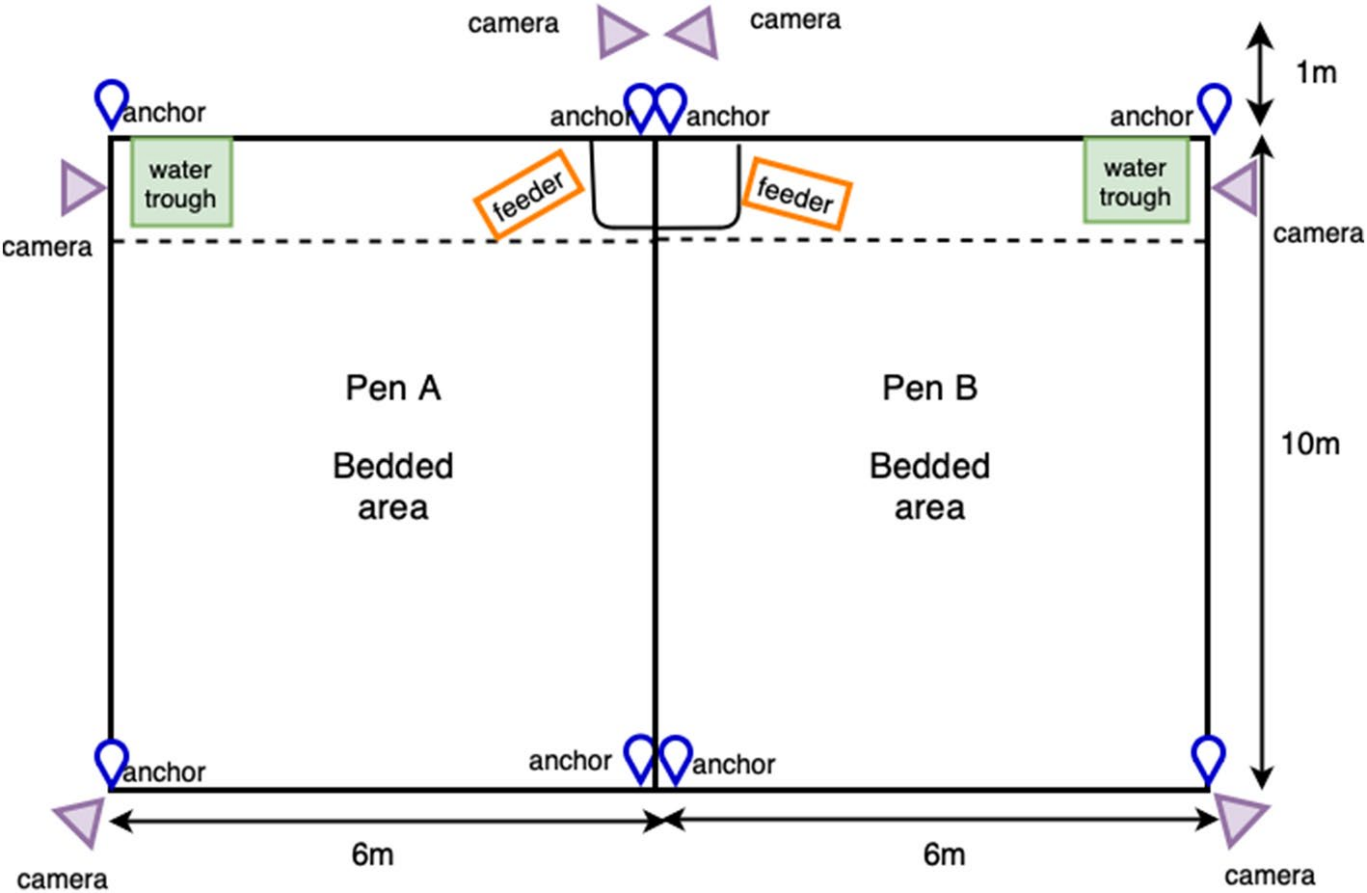
Schematic diagram of the two pens used during this study. Anchors were located at the corners of each of the pens. Feeder, water trough and video cameras are also indicated in the diagram.

The Ultra-wideband Sewio Leonardo iMU tracking sensors (Noldus, Wageningen, the Netherlands)^24^ were attached to each individual calf via a collar with a counterweight to help to maintain the position of the sensors. These UWB sensors served as transmitters sending out blinks to the anchors to calculate the tag position^30^. UWB sensors were able to obtain the position of the calves by using the time difference of the arrival of the RF signals, which provides the distance between the reference point and the sensor^31^. Anchors were used to detect the pulses emitted by the UWB sensors and then send this information to the location server to calculate the location. There were four fixed anchors located at each of the corners of the two pens as described in Fig. 1. The computer with the dedicated software (TrackLab) retrieved and processed all the location data in real-time. The relative local coordinates were automatically generated on the system for each of the sensors and they could be retrieved from a remote connection to the system.

Each tracking sensor logged the relative local coordinates (*x,y*) of each individual animal at a frequency rate of 1 Hz. With this sampling frequency, the battery life of sensors is 5 months on average, which cover the total duration of the calves in the pens and hence it was not necessary to change collars. However, the systems provided a visualisation of the battery life, allowing us to change sensors if required.

A validation test was performed to estimate the precision and accuracy of the location system. The validation provided a mean circular error probability (CEP) of 0.15 m (range 0.12–0.28 m) and DIST of 0.17 (range 0.13–0.33 m), representing the precision and accuracy of the location, respectively, and computed following Barker et al.^32^.

#### Pre-processing and cleaning of positional data

Any periods of time when calf behaviour was affected by human proximity interactions were removed from the initial dataset. Additionally, location data from the first day when calves were moved to the pens were removed. Any periods when data were lost due to power cuts or battery drainage of the tags, and when local coordinates were outside of the pen were also removed from the data. In total 7.98% (444.65 of 5568 total hours) of the data were removed from the 3 cohorts. The position data were smoothed using a moving average over a 10-s to improve the accuracy of the location.

#### Play behaviour labelling

Play behaviour was recorded using three video cameras (HikVision) that were mounted on the walls at an altitude of 3–4 m and oriented to ensure all the pen was covered by them. The cameras were set to record at a frequency of 30 frames/s and resolution of 2944 × 1656 pixels. All the cameras were connected to a 4 MB video recorder from which video recordings were retrieved.

The video footage was labelled by two trained observers. Video recordings were coded into play and non-play behaviours by playing each video and manually annotating the start and stop times in an Excel spreadsheet. All calves were sprayed on either side with a colour spray to facilitate their identification. For each cohort, 10–12 h of video footage was labelled each day for three days. The criteria for selecting which days to label video footage were: (1) when calves were freshly sprayed to ease identification and (2) a few days after moving to a new pen to allow for habituation of calves. In total 101.36 h of play and non-play behaviour were labelled. All instances of locomotion play behaviour were labelled. A detailed description of the number of samples per calf per cohort is provided in the Supplementary Table S1 online. In this study, locomotion play behaviour was defined as rapid forward movement that lasts a minimum of 3 s and could include jumping or bucking instances as previously described in Carslake et al.^19^.

#### Data processing

The behavioural observations and raw location data were merged based on timestamps using custom made scripts written in Matlab^33^. Any delays due to timestamp desynchronisation between the clocks of the video camera recordings and the location system were corrected using video visualisations of trajectory data alongside the associated video camera recordings for specific events such as a calf entering the automatic milk feeder. This allowed us to identify the exact time when the calf enters the automatic milk feeder and annotate the timestamps from the camera recordings and the trajectory video visualisations. From these two timestamps the delay can be computed. The merged files containing both sensor and location data were discretised into windows of 3 s each to compute feature characteristics. This was performed in two steps. First, for each individual window of 3 s the speed, turning angle and turning angle-speed were computed according to the definition and formula shown in Table 2. Second, for these three components (speed, turning angle and turning angle-speed) the following characteristics were computed: mean, standard deviation, sum, maximum and minimum, giving a total of 15 feature characteristics.

**Table 2:** Description, definition and formulas of the measures used to compute feature characteristics.

Measure	Definition	Formula
Speed	Euclidean distance between two consecutive locations divided by time duration between locations	$${\raise0.7ex\hbox{${\sqrt {(x_{{t + 1}} - x_{t} )^{2} + (y_{{t + 1}} - y_{t} )^{2} } }$} \!\mathord{\left/ {\vphantom {{\sqrt {(x_{{t + 1}} - x_{t} )^{2} + (y_{{t + 1}} - y_{t} )^{2} } } {\left( {(t + 1) - t} \right)}}}\right.\kern-\nulldelimiterspace} \!\lower0.7ex\hbox{${\left( {(t + 1) - t} \right)}$}}$$
Turning angle	Angle between direction of movement for successive locations	${cos}^{-1}\frac{(a\cdot b)}{\left|a\right|\left|b\right|}$ where $\cdot$ represents the dot product which can be computed as $a\cdot b={x}_{1}*{x}_{2}+{y}_{1}*{y}_{2}$ with $a =\left({x}_{1},{y}_{1}\right) and b=\left({x}_{2},{y}_{2}\right) .$ Here $\left|a\right|$ represents the norm of the vector and can be computed as $\left|a\right|= \sqrt{{x}_{1}^{2}+{y}_{1}^{2}}$
Turning angle-speed	Product of the speed times the cosine of the turning angle	Speed × (cos(turning angle))

### Classification of play behaviour

**Figure 2:**
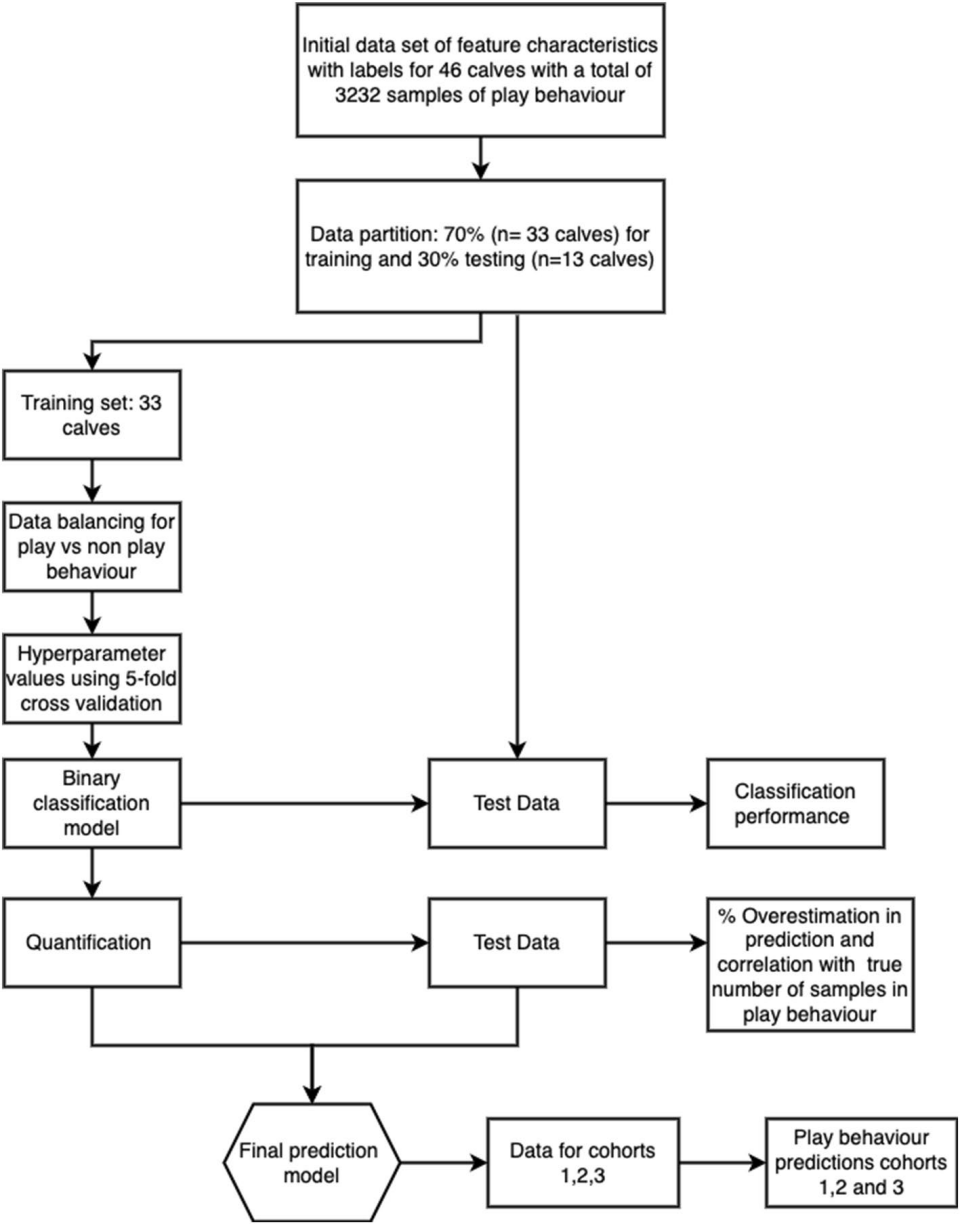
Overview of the process for the classification and quantification of play behaviour.

For the classification of play versus non-play behaviour, a 2-step classification and quantification algorithm was developed as illustrated in Fig. 2. In the first step, a classification algorithm was built over the train set and in the second step this classification algorithm was used to implement an adjusted count quantification algorithm. A schematic diagram of the 2-step classification and quantification algorithm used is shown in Fig. 2. Initially, all the dataset, which consisted of the feature characteristics and labels, was partitioned into training and testing subsets using a 70/30 split. Data were split using stratification by calf identity to ensure that there was no data leakage from the training data into the test dataset^34^. During training, data was balanced using a random under sampling technique^35,36^ to address the interclass imbalance between play (2182 instances of play) and non-play instances (1323 607 instance of non-play). Within the training dataset, an Adaboost ensemble learning algorithm was implemented using the fitcensemble function in Matlab. Selection of the Adaboost learning algorithm was based on its success from its early appearance^37^, recent years^38^ and previous success on classifying locomotor play using accelerometer-based sensors^19^. Moreover, AdaBoost learning algorithm has been shown to be more robust than random forest when applied to large imbalanced datasets^39^. Optimal parameters were obtained using the automatic ‘OptimizeHyperparameters’ tool from Matlab, which uses a Bayesian optimisation over the number of learning cycles, over the learning rate and over the maximum number of splits providing the ensemble classifier with the minimum cross-validation loss. During the optimisation, fivefold cross validation^40^ was utilised to estimate the misclassification rate and to select parameters that minimise it. Optimal parameters were an Adaboost leaner with maximum number of splits of 10, number of learning cycles of 494, and learning rate of 0.001016. The complete list of parameters evaluated during the optimisation is provided in the Supplementary Table S2 online. Classification performance for play versus non-play behaviour was evaluated using a holdout test dataset to avoid any data leakage. Algorithm performance was evaluated using different performance metrics, which included overall accuracy, specificity, sensitivity, precision and F-score as described in Dohoo et al.^41^.

### Quantification algorithm

In the second step of the algorithm, play behaviour was quantified using an adjusted count (AC) technique with a maximum selection threshold as described in Forman^42^ to correct for the tendency of classification algorithms to overestimate when predicting low prevalence behaviours^25,43^. The AC algorithm is based on a binary classification with correction. The AC algorithm first trains a binary classifier and then it corrects the prevalence of an unknown sample to the formula:

$${p}{\prime}=\frac{{p}_{0}{\prime}-fpr}{tpr-fpr}$$

where ${p}_{0}{\prime}$ represents the initial estimate of prevalence from the binary classifier, ${p}{\prime}$ represents the adjusted prevalence and its true positive rate *(tpr)* and false positive rate (*fpr*) are computed as tpr = tp/(tp + fn) and fpr = fp/(tn + fp), respectively, with TP, FN, FP, TN, indicating the true positive, false negative, false positive and true negative, respectively. For the quantification of play behaviour, we used the Adaboost classification algorithm for the binary classification. When working with highly imbalanced datasets, it has been suggested to select a threshold that maximises the denominator in the above formula (tpr-fpr) over a range of different training conditions. A varying range of training conditions were generated by randomly selecting *p* = 10,80,150,…,2180 play instances, and 250,000 non-play instances from the training subset. The *tpr* and *fpr* were computed from each value of the training conditions using a fivefold cross validation over the binary Adaboost classification algorithm. Afterwards, the value of the training condition that maximises *tpr-fpr* in the previous equation was selected to train a binary Adaboost classification algorithm over the train set. The value that maximised *tpr-fpr* was obtained when using 2180 play instances and 250,00 non-play instances. This corrected binary Adaboost classification algorithm was used to predict the number of play instances over the test dataset.

A comparison between the number of observed samples of play behaviour and the number of predicted samples from the quantification algorithm for each individual calf on the test set was performed via non-parametric correlation. The percentage of overestimation of play behaviour was computed as:

$$overestimation =\left(\frac{number \, of \, predicted \, samples-number \, of observed \, samples}{number \, of \, observed \, samples}\right)\times 100$$

where the number of predicted samples was calculated using the test data and the number of observed samples was also based on the labelled test dataset. Overestimation was computed over the whole test dataset.

The final evaluated quantification algorithm was then used to predict play behaviour during the whole period in which calves were housed in the 6 m × 10 m pens, for each of the calves for each day and for all three cohorts. Hence, prediction was performed over the feature characteristics obtained from UWB location data on a 3 s window for each calf for the period in which they were housed in the 6 m × 10 m pens (up to 18 weeks). Two different measures were computed and summarised per day: total amount of play behaviour (in seconds) and number of instances of play behaviour.

### Factors associated with play behaviour

We investigated the impact that age, weaning, health and sex have on the play behaviour by fitting a generalized linear mixed model with a log link function using the lme4 package^44^ in R for the total amount of play behaviour per day and generalized linear mixed model with a non-negative binomial distribution for the number of instances of play behaviour per day. The models were defined as follows:

$$Y = X\beta +Z\alpha +\varepsilon$$

where Y represents each of the play behaviour measures (total of play per day and number of play instances), X represents the fixed models: age of the calf (in days), sex and breed, cohort, weaning stage, mean ambient temperature (average of temperature recordings on a given day), and health status of the calf, and Z represents the random effects: calf ID and $\varepsilon$ represents the residuals. Sex-breed, weaning and health status were all defined as categorical variables with sex-breed having two categories: female HO and male HO × AA, weaning with three categories (feed-milking, step-down and weaned) and health with three categories (healthy, moderate, and sick).

## Results

### Classification

**Figure 3:**
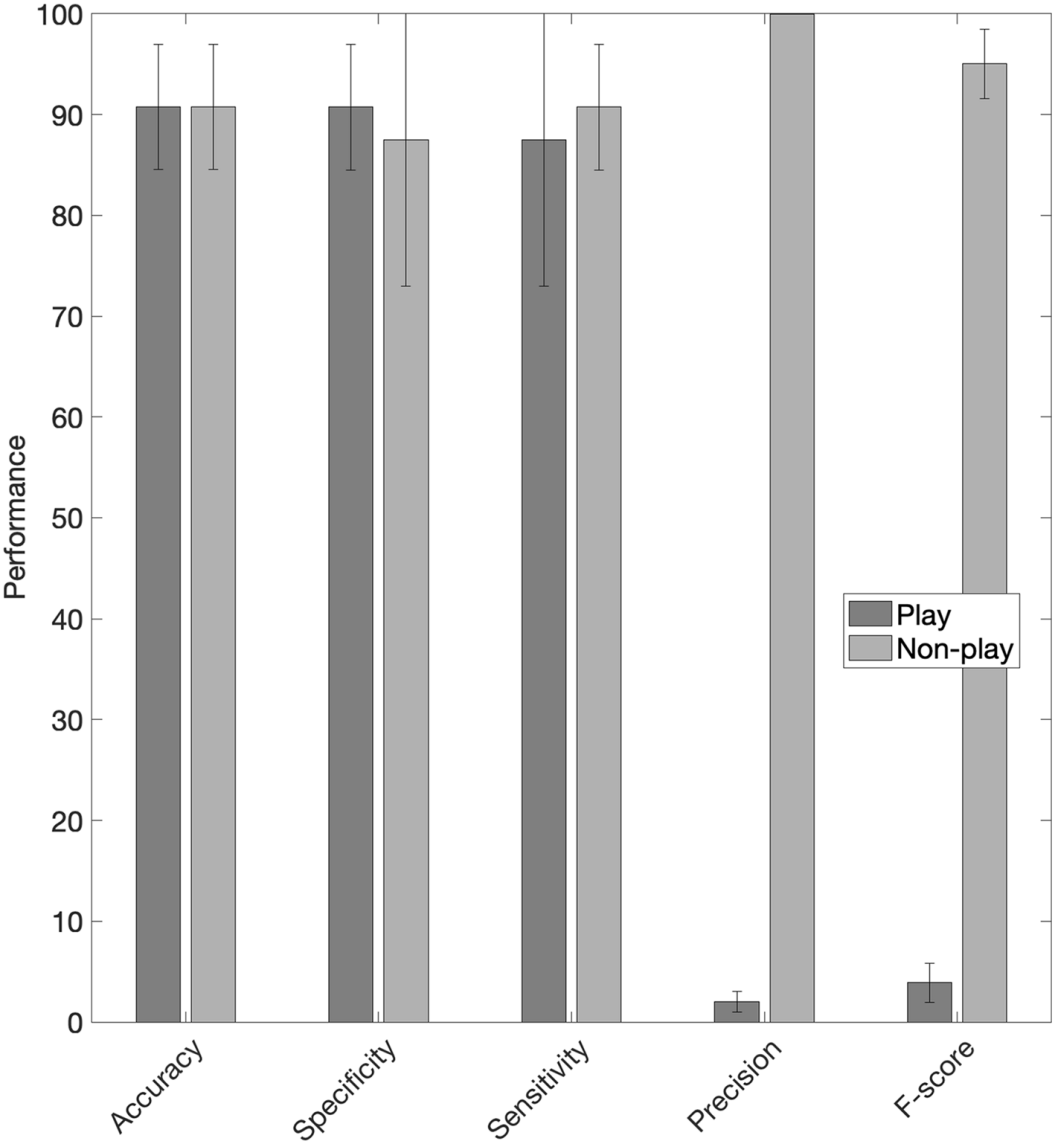
Performance (%) of the classifier for play versus non-play behaviour. The bars show the average performance for all the calves whereas the black error bars show the variation in performance across the different calves.

Results of the classification of play behaviour using the test dataset are shown in Table 3 as well as in Fig. 3.

**Table 3:** Classification performance for play and non-play behaviour for the different cohorts.

Cohort	Behaviour	Accuracy	Specificity	Sensitivity	Precision	F-score
1	Non-play	94.60	96.37	94.60	99.99	97.22
	Play	94.60	94.60	96.37	2.88	5.,60
2	Non-play	96.42	75.42	96.43	99.98	98.18
	Play	96.42	96.43	75.42	1.48	2.90
3	Non-play	84.65	94.99	86.62	99.98	91.66
	Play	84.65	84.62	94.99	1.86	3.66
Overall	Non-play	90.60	93.05	90.59	99.98	95.06
	Play	90.60	90.59	93.05	1.95	3.82
By calf	Non-play	90.57($\pm$ 6.33)	87.57($\pm$ 14.55)	90.56($\pm$ 6.33)	99.98($\pm$ 0.01)	94.93($\pm$ 3.55)
	Play	90.57($\pm$ 6.33)	90.56($\pm$ 6.33)	87.52($\pm$ 14.55)	1.97($\pm$ 1)	3.83($\pm$ 1.91)

### Quantification results

**Figure 4:**
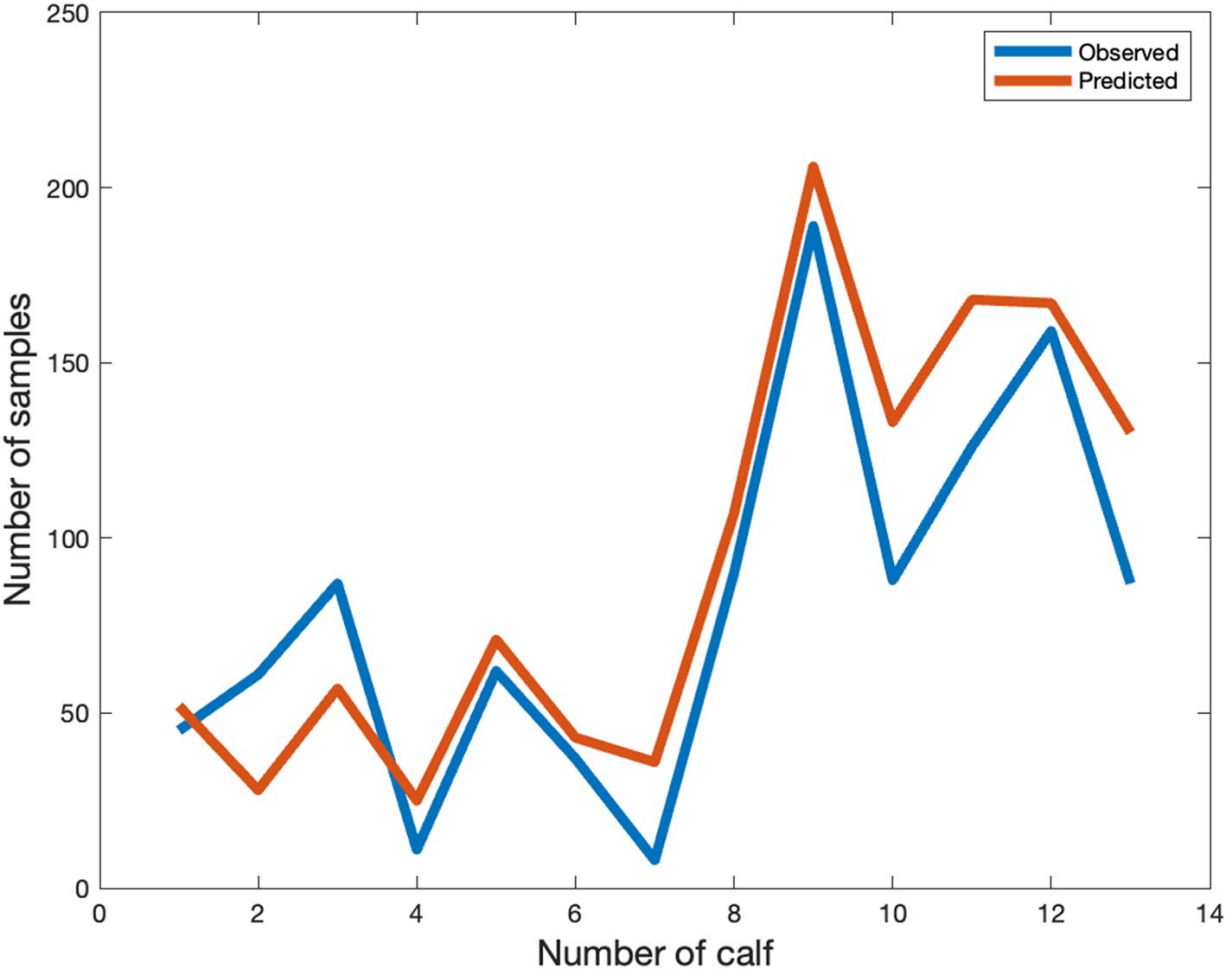
Comparison of the number of samples observed as play versus the number of samples predicted as play using the AC quantification algorithm for each of the individual datasets. A datafile number represents the number of days that were labelled.

Quantification of play behaviour using the AC method is shown in Fig. 4 where the number of samples predicted as play versus the number of samples observed play for each of the datafiles is shown. A significant positive correlation of 0.9125 (*p*-value < 0.0034) was obtained between the predicted number of play and the observed number of play samples. The total number of samples from which 1223 were predicted as play versus 1050 observed samples of play, providing an overestimation of 173 samples (16.48.%).

### Temporal variation of play behaviour

**Figure 5:**
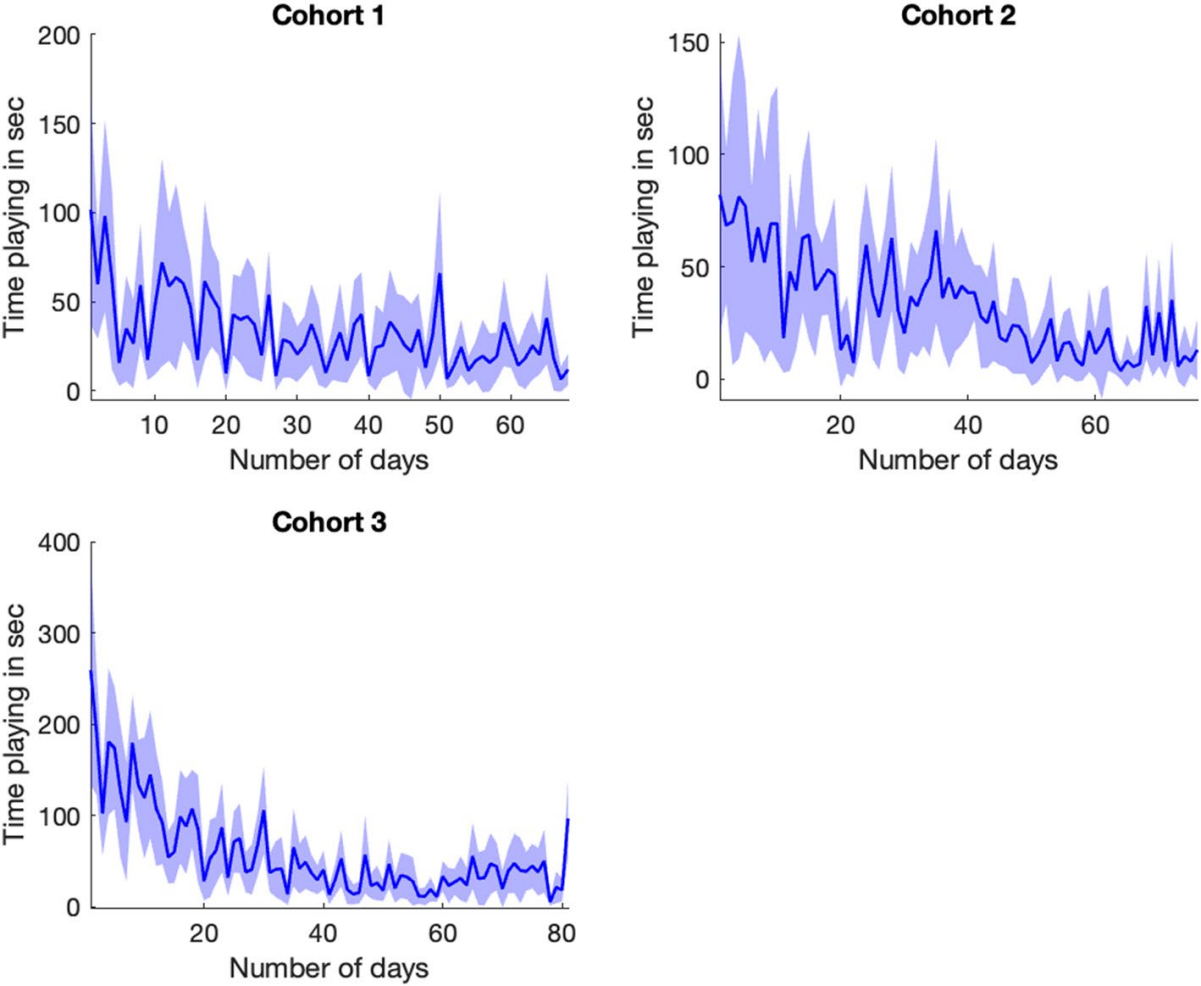
Temporal variation of the total amount (in seconds) of play behaviour per calf for all different cohorts. Dark blue line represents the mean across calves, the light blue shows the mean $\pm$ one standard deviation.

The total amount and the number of instances of play behaviour per calf per day were obtained using the quantification technique. Figure 5 shows temporal variation of the total amount of play per calf per day over the three different cohorts in this study.

### Association between age, individual health/weaning and play behaviour

#### Model for the total amount of play behaviour

The results from the glm model showed that increasing the age by one day significantly decreased total playing time by 63% (Table 4). Compared with cohort 1, cohort 2 had a significantly higher total play time by 125%. Compared to pre-weaned calves, the total play time significantly increased by 30% for calves in the step-down weaning process and significantly increased by 145% when completely weaned. Sick calves had significantly less play time by 14% compared to healthy calves. The total amount of variability explained by calf ID was 13.88% on the total playing time. We also implemented the same model for total play time removing the 3 male HO X AA calves and qualitatively similar results were obtained.

**Table 4:** Effect of age, cohort, weaning, health status, temperature, sex and breed in the total amount of play behaviour per day.

	N	Estimate	Std error	*P*-value	Exp(weight)
Intercept	3451	2 2.86	0.12	< 0.0001	
Age	3451	− 0.98	0.03	< 0.0001	0.37
Cohort 1	1020	Ref			
Cohort 2	1216	− 0.10	0.15	0.4832	0.90
Cohort 3	1215	0.80	0.15	< 0.0001	2.25
Pre-weaned	1625	Ref			
Weaning stepdown	920	0.27	0.06	< 0.0001	1.30
Weaned	906	0.90	80.08	< 0.0001	2.45
Health status healthy	3194	Ref			
Health status sick	240	− 0.14	60.06	0.0328	0.86
Female HO	3451	Ref			
Male HO x AA	204	− 0.19	0.25	0.46	1.20
Mean Temperature	3451	− 0.04	0.030	0.11	1.04

#### Model for the number of instances of play behaviour

The results from the glm model showed that increasing the age by one day significantly decreased the number of playing instances by 2% (Table 5). Compared with cohort 1, cohort 3 had a significantly higher number of playing instances by 63%. Compared to pre-weaned calves, the number of playing instances significantly increased by 10% for calves in the step-down weaning process and significantly increased by 52% when completely weaned. Sick calves had significantly lower number of playing instances by 12% compared to healthy calves. An increase in the mean daily temperature significantly decreased the number of instances of play by 1%. The total amount of variability explained on the number of instances of play by calf ID was 12.27%. We also implemented the same model for the number of instances removing the 3 male HO X AA calves and qualitatively similar results were obtained.

**Table 5:** Effect of age, cohort, weaning, health status, temperature, sex and breed in the number of instances of play behaviour per day.

	N	Estimate	Std error	*P*-value	Exp(weight)
Intercept	3451	4.04	0.13	< 0.0001	
Age	3451	− 0.02	0.001	< 0.0001	0.98
Cohort 1	1020	Ref			
Cohort 2	1216	− 0.09	0.10	0.3665	0.91
Cohort 3	1215	0.49	0.10	< 0.0001	1.63
Feed-milking	1625	Ref			
Weaning stepdown	920	0.10	0.04	0.0298	1.10
Weaned	906	0.42	0.06	< 0.0001	1.52
Health status healthy	3194	Ref			
Health status sick	240	− 0.12	0.05	0.0135	0.88
Female HO	3451	Ref			
Male HO × AA	204	− 0.18	0.17	0.2719	0.83
Mean temperature	3451	− 0.008	0.003	0.0379	0.99

## Discussion

To the authors’ knowledge, this is the first study to use UWB location data to measure and monitor locomotor play behaviour of farmed calves. The adjusted count quantification algorithm that we used to predict play behaviour had significantly lower overestimation of 16% compared to previous play algorithms, which reported overestimation of 200%^18^. Moreover, utilising predictions from the quantification method, we were able to investigate the impact that different factors, such as age, individual health status, and weaning status, had on the daily amount of play behaviour and the daily number of play behaviour instances. The ability to detect and quantify play behaviour in farmed calves is important for the development of objective measures of positive welfare, which are currently lacking despite increased interest in this topic^6^. The use of automated data collection and quantification allowed long-term monitoring of a large number of individuals experiencing different health and weaning stages, which would have been impractical and time consuming otherwise.

Our classification algorithm for play behaviour performed well, with values > 90% across most of the different performance metrics (accuracy, specificity, and sensitivity). The data split was stratified by calf ID, preventing data leakage of information from the same calf to be contained in both the training data and the test dataset, which would lead to inaccurate predictions that can be overly optimistic^34^. Data leakage is a widespread problem in machine learning, which can limit the reproducibility of these methods^45^. Our results show that the algorithm was robust since standard deviation on the performance for the different calves was relatively low with 6.33% for accuracy, 6.33% for sensitivity, 14.55% for specificity, 1% for precision, 1.91% for F-score. It is important to highlight that the classification algorithm had low precision (1.95%), which represented a high number of false positives for predicting play behaviour. This is a common problem for low prevalence behaviours^19^ and shows the need for the use of quantification methods to correct for the overestimation.

To correct for the tendency of classification algorithms to provide overestimations when predicting behaviours that are of low prevalence^18^, we used an adjusted count quantification method which overestimated play behaviour by only 16.48%. This was a slight improvement on a previous study, which overestimated play behaviour by 19% and used an adjusted count quantification method to predict play behaviour from accelerometer data^19^. This suggests that play behaviour can be predicted using automated location data. Location data has also been successfully used to predict personality in dairy calves^24^ and lying behaviour of dairy cows^23^. Our results suggest that our quantification method could be used to detect other low prevalence behaviours from location data. Consequently, there is potential for location data to be used to develop other indicators of positive welfare, such as grooming with a brush^46^.

Our study was novel in that we were able to follow calves for a period of up to 18 weeks, whereas previous studies using sensors measured calf behaviour for a shorter period ranging from 48 h to 12 days^19,47^. Our longer monitoring period enabled us to understand how play behaviour changes over time, including during the step-down and weaned period, as well as the impact of health status. The results show that the total daily play time and instances of play per day significantly reduced with age. This result is similar to previous studies which measured play behaviour using observational methods^8,48^. However, these studies only observed calves for 24–48 h at 3–6 time points in the calves’ life. Our study strengthens the conclusion that play behaviour decreases with age as we monitored calves’ behaviour continuously over 18 weeks.

Whilst play behaviour decreased with age, our results also showed that play behaviour significantly increased during the step-down weaning process and increased even more after weaning (Table 4). This result is in contrast with previous research which indicated that play behaviour was reduced post-weaning and is linked to energy intake^8,49^. Additionally, Jensen and Kyhn^48^ suggested that the decrease in play behaviour as calves age was likely due to an effect of weaning. Our results did not support this suggestion, as they indicated that the decrease in play behaviour with age was not an effect of weaning. The inclusion of age and weaning in our models enabled us to understand the effect of weaning whilst controlling for age. It should be noted that our results may differ from previous research due to different weaning methods. Here, we used a step-down weaning process over a period of 20 days, which is associated with more solid feed intake and gains in bodyweight^50^. In contrast, previous studies have compared and used different methods of weaning including shorter weaning periods and different levels of milk^8^ and grain consumption^49^. In the present study, we adopted a longer, more gradual weaning period than Krachun et al.^8^ or Jensen and Kyhn^48^. As weaning is a stressful event that can cause a strong behaviour response^51,52^, it is possible that the difference in results may be due to a difference in stress caused by weaning.

Calves that were categorised as sick had significantly decreased total play time by 14% and instances of play by 12%. Größbacher et al.^53^ also showed that health impairments reduced the instances of play; however, the total play time was not reduced in their study. In another study, calves’ health status did not affect their own play behaviour, but did affect their playmates^54^. It should be noted that the calves were housed in pairs in the study by Bertelsen and Jensen^54^, in groups of 1–3 in Größbacher et al.^53^, and in groups of at least fifteen in our study, and this might be a reason for differing results. Together these results suggest that play behaviour may be an indicator of calf health; however, this may also be impacted by the social environment. For example, it is known that increases in space allowance^48^ and social contact^55,56^ also increase play behaviour in calves.

Calves in cohort 3 played for significantly longer times per day and had significantly more instances of play per day. This suggests that the individual variation of calves in these cohorts could be different. Indeed, the amount of variability explained by calf ID (12.27%) in the number of instances of play highlights that there is a level of individual difference in play behaviour, and this may be explained by a variety of factors including personality^24,57^, sex and breed^58^. We should note that cohort 2 had lower sensitivity for play compared to the other two cohorts. We believe that this may be due to calves in cohort 2 being younger than the other cohorts by approximately five days, which may affect the speed of their playing behaviour. Thus, the impact of calf-level variables on play behaviour could be investigated in future studies.

Our study also shows that the physical environment has an impact on play behaviour in calves. Increased mean ambient temperature significantly decreased the number of instances of play by 1%. One previous study has also shown that the duration of play behaviour in calves decreases with higher maximum daily ambient temperatures^53^. It is important to consider that we have used location data to predict play behaviour in one highly controlled environment. Therefore, further studies are required to investigate whether our method can be applied in different environments with similar results. We would also like to highlight that we did not measure the agreement between the two observers who were labelling the play behaviour. However, both observers were trained by the same individual. Furthermore, play is a distinctive behaviour, so there is likely to be a low degree of subjectivity between observers.

In conclusion, this study shows that UWB location data can be used to accurately and precisely predict and quantify and monitor locomotor play behaviour in farmed calves, whilst correcting for overestimation. Our results indicate that the instances and duration of play behaviour per day significantly decreased with age and when calves were categorised as sick, whilst play behaviour significantly increased during the weaning stage and even more after weaning. This study shows that precision livestock technologies such as location sensors can be used to monitor positive welfare longitudinally by detecting low prevalence behaviours such as play behaviour.

### Supplementary Information

Supplementary Tables.

## Data Availability

The datasets supporting the results of this article are unavailable due to contractual reasons but may be available upon reasonable request from the corresponding author.
