# Supplementary material for: Quantification of play behaviour in calves using automated ultra-wideband location data and its association with age, weaning and health status
Source: Sci Rep. 2024 Apr 17;14:8872. doi: 10.1038/s41598-024-59142-z (PMC11024191; doi:10.1038/s41598-024-59142-z)
Supplement: Supplementary file 1 — Supplementary Tables. [file 41598_2024_59142_MOESM1_ESM.docx]

Quantification of play behaviour in farmed calves using automated ultra-wide band location data and its association with age, weaning and health status

Vázquez-Diosdado, J.A^1^., Doidge, C.^1^, Bushby, E.V.^1^, Occhiuto, F.^1^, & Kaler, J.^1^ *

^1^ School of Veterinary Medicine and Science, Sutton Bonington Campus, University of Nottingham, Leicestershire, LE12 5RD, UK.

**Supplementary Material**

Table 1. Description of the number of play and non-play instances labelled for each calf on the three different cohorts.

| Calf ID | Cohort | Number of samples of play | Number of samples of non-play | Original Calf ID |
| --- | --- | --- | --- | --- |
| 1 | 1 | 18 | 38688 | 3808 |
| 2 | 1 | 45 | 38663 | 3811 |
| 3 | 1 | 44 | 38662 | 3812 |
| 4 | 1 | 35 | 38671 | 3813 |
| 5 | 1 | 9 | 38699 | 3814 |
| 6 | 1 | 27 | 38679 | 3820 |
| 7 | 1 | 11 | 38695 | 3822 |
| 8 | 1 | 61 | 38647 | 3826 |
| 9 | 1 | 91 | 38615 | 3827 |
| 10 | 1 | 73 | 38633 | 3828 |
| 11 | 1 | 47 | 38661 | 3829 |
| 12 | 1 | 98 | 38608 | 3831 |
| 13 | 1 | 61 | 38645 | 3833 |
| 14 | 1 | 10 | 38698 | 3834 |
| 15 | 1 | 87 | 38619 | 3835 |
| 16 | 2 | 26 | 41616 | 3853 |
| 17 | 2 | 27 | 41617 | 3854 |
| 18 | 2 | 11 | 41631 | 3856 |
| 19 | 2 | 27 | 41615 | 3859 |
| 20 | 2 | 25 | 41619 | 3862 |

| Calf ID | Cohort | Number of samples of play | Number of samples of non-play | Original Calf ID |
| --- | --- | --- | --- | --- |
| 21 | 2 | 8 | 41634 | 3863 |
| 22 | 2 | 43 | 41599 | 3864 |
| 23 | 2 | 14 | 41630 | 3865 |
| 24 | 2 | 4 | 41638 | 3868 |
| 25 | 2 | 62 | 41580 | 3871 |
| 26 | 2 | 37 | 41607 | 3873 |
| 27 | 2 | 91 | 41551 | 3875 |
| 28 | 2 | 0 | 41642 | 3876 |
| 29 | 2 | 68 | 41576 | 3878 |
| 30 | 2 | 4 | 41638 | 3880 |
| 31 | 2 | 8 | 41634 | 3886 |
| 32 | 3 | 90 | 40088 | 4355 |
| 33 | 3 | 87 | 40090 | 4359 |
| 34 | 3 | 136 | 40042 | 4360 |
| 35 | 3 | 189 | 39989 | 4362 |
| 36 | 3 | 88 | 40089 | 4363 |
| 37 | 3 | 126 | 40052 | 4364 |
| 38 | 3 | 170 | 40008 | 4367 |
| 39 | 3 | 159 | 40018 | 4368 |
| 40 | 3 | 238 | 39940 | 4370 |
| 41 | 3 | 180 | 39998 | 4374 |
| 42 | 3 | 59 | 40118 | 4375 |
| 43 | 3 | 202 | 39976 | 4377 |
| 44 | 3 | 116 | 40062 | 4378 |
| 45 | 3 | 133 | 40044 | 4381 |
| 46 | 3 | 87 | 40091 | 4382 |

Table 2. Set of parameters evaluated using a Bayesian optimisation with 5-fold cross validation. In bold font is the set of parameters that provide the best results using the objective function (loss function).

| Iter | Objective | BestSoFar | NumlearningCycles | Learning Rate | MaxNumSplits |
| --- | --- | --- | --- | --- | --- |
| 1 | 0.09945 | 0.09945 | 91 | 0.60549 | 319 |
| 2 | 0.10243 | 0.09945 | 340 | 0.0038563 | 357 |
| 3 | 0.06989 | 0.06989 | 137 | 0.31479 | 2 |
| 4 | 0.10403 | 0.06989 | 34 | 0.0040919 | 1871 |
| 5 | 0.072869 | 0.06989 | 10 | 0.0011649 | 1 |
| 6 | 0.071952 | 0.06989 | 11 | 0.93762 | 5 |
| 7 | 0.07264 | 0.06989 | 14 | 0.98981 | 1 |
| 8 | 0.065765 | 0.065765 | 498 | 0.059979 | 3 |
| 9 | 0.071036 | 0.065765 | 486 | 0.431 | 9 |
| 10 | 0.066682 | 0.065765 | 499 | 0.076129 | 3 |
| 11 | 0.068515 | 0.065765 | 500 | 0.24186 | 3 |
| 12 | 0.064849 | 0.064849 | 490 | 0.049555 | 3 |
| 13 | 0.065995 | 0.064849 | 374 | 0.0033918 | 6 |
| 14 | 0.065995 | 0.064849 | 452 | 0.013234 | 4 |
| 15 | 0.067828 | 0.064849 | 17 | 0.015687 | 4 |
| 16 | 0.066224 | 0.064849 | 467 | 0.0011177 | 4 |
| 17 | 0.065995 | 0.064849 | 491 | 0.009193 | 4 |
| 18 | 0.065078 | 0.064849 | 489 | 0.0039411 | 3 |
| 19 | 0.065307 | 0.064849 | 498 | 0.007007 | 3 |
| 20 | 0.098533 | 0.064849 | 337 | 0.91336 | 1 |
| 21 | 0.07516 | 0.064849 | 10 | 0.0012767 | 42 |
| 22 | 0.065307 | 0.064849 | 488 | 0.0080182 | 3 |
| 23 | 0.072869 | 0.064849 | 476 | 0.93712 | 56 |
| 24 | 0.10357 | 0.064849 | 18 | 0.0011596 | 4359 |
| 25 | 0.065307 | 0.064849 | 495 | 0.00414 | 3 |
| 26 | 0.065078 | 0.064849 | 447 | 0.0010846 | 11 |
| 27 | **0.06462** | **0.06462** | **494** | **0.001016** | **10** |
| 28 | 0.065307 | 0.06462 | 488 | 0.0039004 | 15 |
| 29 | 0.065307 | 0.06462 | 461 | 0.0010083 | 13 |
| 30 | 0.065765 | 0.06462 | 484 | 0.0019178 | 11 |
